# Characterizing the differences between multisystem inflammatory syndrome in children and Kawasaki disease

**DOI:** 10.1038/s41598-021-93389-0

**Published:** 2021-07-05

**Authors:** Maskit Bar-Meir, Alex Guri, Max E. Godfrey, Avram R. Shack, Philip J. Hashkes, Ofra Goldzweig, Orli Megged

**Affiliations:** 1 Pediatric Infectious Diseases, Shaare-Zedek Medical Center, P.O.B 3235, 91301 Jerusalem, Israel; 2 The Faculty of Medicine, Hebrew University, Jerusalem, Israel; 3 Pediatric Department, Kaplan Medical Center, Rehovot, Israel; 4 Pediatric Department, Shaare-Zedek Medical Center, Jerusalem, Israel; 5 Pediatric Rheumatology Unit, Shaare-Zedek Medical Center, Jerusalem, Israel; 6 Pediatric Rheumatology Unit, Kaplan Medical Center, Rehovot, Israel; 7 Pediatric Cardiology Unit, Shaare-Zedek Medical Center, Jerusalem, Israel

**Keywords:** Fever, Skin manifestations, Risk factors, Infectious diseases

## Abstract

To characterize the new SARS-Co-V-2 related multisystem inflammatory syndrome in children (MIS-C) among Israeli children and to compare it with Kawasaki disease (KD). We compared, in two medical centers, the clinical and laboratory characteristics of MIS-C, KD and an intermediate group, which met the case definitions of both conditions. MIS-C patients were older, were more likely to be hypotensive, to have significant gastrointestinal symptoms, lymphopenia and thrombocytopenia and to have non-coronary abnormal findings in their echocardiogram. Lymphopenia was an independent predictor of MIS-C. Most of our MIS-C patients responded promptly to corticosteroid therapy. KD incidence in both centers was similar in 2019 and 2020. Although there is clinical overlap between KD and MIS-C, these are separate entities. Lymphopenia clearly differentiates between these entities. MIS-C patients may benefit from corticosteroids as first-line therapy.

## Introduction

Soon after the COVID-19 pandemic started, a new entity—termed multisystem inflammatory syndrome in children (MIS-C; also referred to as pediatric inflammatory multisystem syndrome, PIMS) was reported from many countries^1–5^. Separate case definitions were developed by the Centers for Disease Control and Prevention (CDC) and the World Health Organization (WHO)^6,7^. Both sets require fever, elevated inflammatory markers and involvement of at least two systems, with evidence of prior SARS-CoV-2 infection or exposure, as well as exclusion of other potential etiologies. Since Kawasaki disease (KD) shares some of the clinical and laboratory features with this new entity (fever, rash, conjunctival injection, elevated inflammatory markers), and both rely for their diagnosis on a set of non-specific criteria, it was suggested that both KD and MIS-C are in fact part of the spectrum of SARS-CoV-2-related inflammatory illness^2,8^. This question has prognostic as well as therapeutic implications. While in KD the initial standard of care requires a single dose of intravenous immune globulin (IVIG)^9^, the optimal management of patients meeting the MIS-C criteria is unclear. Per published guidelines both IVIG and corticosteroids may be used as first-line therapies, either alone or in combination with biologic agents like anakinra (recombinant human IL-1 receptor antagonist) or tocilizumab (humanized monoclonal antibody against IL-6 receptor)^10^. Our aim is to report our experience during the COVID-19 pandemic with pediatric patients presenting with fever and inflammation (without known etiology) and characterize features that may help differentiating KD from MIS-C.

## Methods

The first case of COVID-19 in Israel was recorded in February 21, 2020. We have collected data of all pediatric patients, meeting the CDC and/or WHO case definitions for MIS-C, who presented to two medical centers serving Jerusalem and the Southern plains of Israel. The Jerusalem area had the highest incidence of COVID-19 in the country^11^. In the Jerusalem medical center we also collected data of all patients who met the KD diagnostic algorithm^9^ during the COVID-19 epidemic. SARS-Cov-2 RT-PCR on nasal swabs was performed for all febrile patients requiring admission. Serology was performed using a commercial kit, which detects IgG against nucleoprotein (Abbott SARS-CoV-2 IgG, Abbott Diagnostics, Abbott Park, IL, USA). The incidence rate of KD was calculated as the number of KD patients, seen in both centers, between January 2019 and October 2020, adjusted per 1000 admissions. During the first lockdown in Israel beginning in March 2020, the number of pediatric admissions dropped by > 60%, therefore for March through June we used the admission numbers of March-June 2019 in each center as the denominator for adjusting KD rates.

The Shaare-Zedek Medical Center (SZMC) and Kaplan Medical Center (KMC) Helsinki Committees have approved this study and its experimental protocol (approval #459/20).

The research was performed in full accordance with relevant guidelines and regulations. Informed consent was waived since all patient data were de-identified.

Analysis of variance (ANOVA) was used to determine whether age, temperature, length of stay in the hospital and mean values of the laboratory tests differed between MIS-C, KD or MIS-C/KD patients. Chi-square test was used to compare the categorical variables between the groups. For all analyses, a 2-sided probability < 0.05 was considered statistically significant. To identify independent predictors for MIS-C, logistic regression model was constructed with MIS-C as the dependent variable (MIS-C vs. KD or MIS-C/KD patients). Variables selected by the univariable analyses were included as independent variables. All analyses were done using SPSS version 25.0.

## Results

Between February 2020 to October 2020, there were 13 patients with KD, 10 with MIS-C and 5 who met both MIS-C and KD case definitions. The clinical and demographic characteristics of the cohort are presented in Table 1.

**Table 1:** Clinical and laboratory characteristics of patients with Kawasaki disease (KD), Multisystem Inflammatory Syndrome in children (MIS-C) and of patients meeting criteria for both conditions.

	Meets KD guidelines, N = 13	Meets both KD and MIS-C guidelines, N = 5	Meets MIS-C guidelines, N = 10	*p*
**Demographic characteristics**				
Median age, months (range)	18 (5–36)	36 (24–193)	136 (60–204)	0.0001
Gender, % male	54	20	60	0.3
Ethnicity				
Jew	9	5	8	
Arab	4	0	2	0.3
**Clinical characteristics**				
SARS-CoV-2				
RT-PCR	0	0	3	
Positive serology	0	2	8	
Exposure	0	3	10	
Fever, mean temperature °C ± SD	39.5 ± 0.4	38.7 ± 0.8	39.4 ± 0.6	0.08
Days of fever ± SD	5.8 ± 2	4.4 ± 2	3.7 ± 1	0.02
Low blood pressure, %*	0	60	60	0.003
Acute gastrointestinal symptoms, %	15	40	90	0.01
Median length of stay in hospital, days (range)	4(3–12)	7(2–13)	7.5(4–21)	0.05
Anti-inflammatory treatment (N of patients receiving)				
IVIG, 2 g/kg	13	5	3	
High dose IV corticosteroids^#^	1	4	10	
IV pulse corticosteroids^##^			1	
Anakinra			3	
Intensive care unit admission	0	2	6	0.006
**Laboratory tests**^**§**^				
White blood count (× 10^3^/µL) ± SD	19 ± 13	15 ± 7	7 ± 5	0.05
Lymphocyte count (× 10^3^/µL) ± SD	5.6 ± 4	1.5 ± 1	0.7 ± 0.5	0.001
Hemoglobin, g% ± SD	10.3 ± 0.9	11.7 ± 1	11 ± 1	0.02
Platelet count (× 10^3^/uL) ± SD	518 ± 365	260 ± 109	136 ± 81	0.006
Na, mEq/L	135 ± 1.8	134 ± 3	134 ± 4.5	0.5
Alanine aminotransferase, IU/L ± SD	62 ± 71	80 ± 119	52 ± 46	0.7
Albumin, g/L ± SD	3.6 ± 0.4	3.0 ± 0.5	3.1 ± 0.6	0.06
C-reactive protein, mg/dL ± SD	13 ± 7	16 ± 4	18 ± 8	0.3
Lactate dehydrogenase umole/L ± SD	298 ± 69	284 ± 317	301 ± 66	0.7
Troponin, ng/L ± SD (nL < 20)	< 6 (N = 1)	4731 ± 9326 (N = 4)	259 ± 526	0.1
D-dimer, ng/mL ± SD (nL < 500)	1638 ± 108 (N = 2)	1455 ± 2511 (N = 3)	2372 ± 2220	0.8
Fibrinogen, mg/dL (nL < 500)	952 (N = 1)	699 ± 187 (N = 3)	688 ± 212	0.5
Ferritin, ng/mL (nL < 205)	235 ± 135 (N = 3)	703 ± 74 (N = 3)	887 ± 1234	0.6
B-type natriuretic peptide (BNP), pg/mL (nL < 100)			799 ± 37	
IL-6, pg/mL (nL < 7)			317 ± 243	
**Echocardiography findings**				
Coronary dilation**	4	2	0	0.09
Decreased LV function	0	0	6	0.004
Valvular regurgitation	2	4	5	0.02
Pericardial effusion	0	1	5	0.009
Retrograde aortic diastolic flow	2	1	5	0.01

*IVIG* intravenous immunoglobulins, *LV*-left ventricular.

^§^Laboratory values are from the day of admission. Median days of illness for the laboratory values were: 5 (± 1.9) for KD patients, 3(± 1.6) for MIS-C patients and 4(± 3) for patients who met both sets of criteria, (the first day of fever considered as day #1 of illness).

*Low blood pressure according to norms for age.

^#^IV Methylprednisolone 2 mg/kg.

^##^IV Methylprednisolone 30 mg/kg.

**3 KD patients with median left anterior descending artery (LAD) Z score of 2.8 (2.2–2.8), 1 KD patient with right coronary artery (RCA) Z-score of 2.8; 2 patients with both KD and MIS-C with LAD Z-score of 2.4 and 2.6 and RCA Z-score of 2.2 and 2.

*MIS-C* patients were predominantly male, aged 5 to 17 years. Eight had positive SARS CoV-2 serology, 3 had positive RT-PCR and all had known exposure to COVID-19. All patients had asymptomatic or mildly symptomatic prior COVID-19 infection. Median time from COVID-19 infection (when known) to presenting with MIS-C and/or KD was 25 days (17–90). One patient without cardiovascular symptoms had severe headache with magnetic resonance imaging (MRI) showing a cytotoxic lesion in the corpus callosum, which was interpreted as a non-specific finding^12^. Most patients (8/10) had acute gastrointestinal (GI) symptoms—all had significant abdominal pain (one had acute abdomen and undergone laparotomy) and 3 with diarrhea. All patients had clinical signs that could resemble those of KD, mainly red eyes and rash (8 and 6 patients, respectively), but none had > 3 clinical KD criteria, and none met the criteria for incomplete KD. Eight of the 10 patients were admitted to the intensive care unit. All received corticosteroid therapy (initially IV methylprednisolone 2 mg/kg/day) with 3 receiving it in conjunction with IVIG. Overall, 70% (7/10) responded promptly, with defervescence of fever after 1–4 doses of corticosteroids. Three patients had continued fever and inflammation and received pulse methylprednisolone (30 mg/kg/day for 3 days) combined with anakinra. All MIS-C patients were well after median follow up of 29 days (7–105). All were successfully weaned from corticosteroids, had normal acute phase reactants and did not develop coronary artery aneurysms (CAA).

### KD patients

Seven (54%) patients had complete KD, the rest met the criteria for incomplete KD. None had myocardial involvement or hypotension. All responded to treatment with IVIG, with 4/13 (30%) requiring a second dose. None developed CAA after median follow up of 23 days (8–115).

### Patients meeting both KD and MIS-C criteria

Five patients met both KD criteria and MIS-C. Of these, 4 had coronary artery dilation and 3 had valvular regurgitation. One 16 year-old patient had all 5 typical clinical KD signs, but also had lymphopenia, thrombocytopenia and excessively elevated troponin. His echo showed left ventricular (LV) dysfunction with no coronary involvement. Of note, he had negative SARS-CoV-2 PCR and serology but resided in an area with a very high incidence of COVID-19. He defervesced after 2 doses of IVIG combined with 2 mg/kg/day of methylprednisolone. A follow-up cardiac MRI demonstrated an area of myocardial scar tissue. Two patients developed uveitis after hospital discharge and required systemic or topical steroids. Over a follow up of 1–2 weeks after discharge, none have developed CAA.

#### MIS-C vs. KD

Several characteristics differentiated MIS-C patients from those with KD: MIS-C patients were older, were more likely to be hypotensive and have significant GI symptoms, have lymphopenia and thrombocytopenia, and have non-coronary abnormal findings in their echocardiogram (LV dysfunction, valvular regurgitation, pericardial effusion or retrograde aortic diastolic flow). Interestingly, the “intermediate” group of patients, who met both sets of criteria, had intermediate values for most clinical and laboratory variables (Table 1, Fig. S1). In multivariate analysis lymphopenia (lymphocyte count < 1500 µL) was an independent predictor of MIS-C, with an adjusted odds ratio of 24 (95% CI 1.3–326, p = 0.02).

#### KD incidence

**Figure 1:**
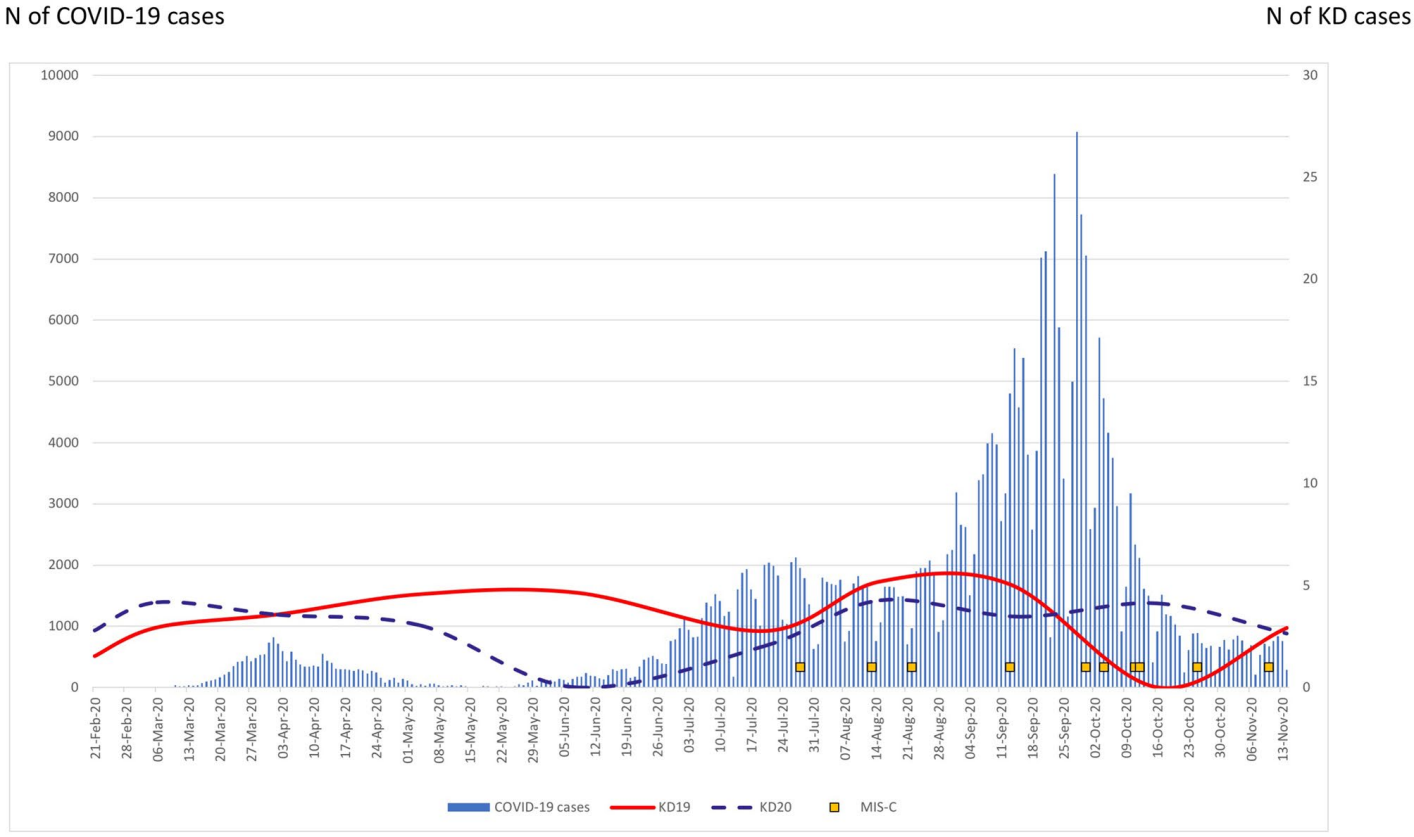
Kawasaki Disease (KD) rates per 1000 admissions in the two medical centers, in 2019 (solid line) and 2020 (dashed line), compared with number of COVID-19 cases diagnosed in Israel (bars; COVID-19 numbers obtained from Israel Ministry of Health data at: www.data.gov.il). multisystem inflammatory syndrome in children (MIS-C) cases represented by squares.

The overall rate of KD admissions in 2019 was 4.8 and 1.2 cases/1000 admissions in SZMC and KMC, respectively, compared with 4.7 and 0.9 in 2020 (calculated until the end of October 2020 and adjusted for the decrease in admissions during the lockdown). Seasonality followed data previously reported from Israel^13^ (Fig. 1).

## Discussion

Our report provides further support from Israeli patients to the new entity of MIS-C, witnessed in many countries, and further characterizes its distinction from the well-defined KD. There is a phenotypic overlap between MIS-C and KD. In particular, considerable similarities exist between MIS-C and KD shock syndrome (KDSS), which is characterized by prominent cardiovascular involvement^2,5^. However, several distinctions help to differentiate these conditions. MIS-C patients were older, had prominent GI symptoms and had lymphopenia and thrombocytopenia, which are not common in KDSS. In fact, we found that lymphopenia was an independent predictor of MIS-C. The clinical presentation in our patients is in line with the MIS-C case series reported so far^1–5^. These reports enrolled patients with positive viral RNA RT-PCR and respiratory symptoms as well as patients with positive serology and/or exposure to COVID-19 patients. Therefore it is possible that some case series represent a mix of patients with acute SARS-CoV-2 infection along with patients with post-infectious inflammation. Furthermore, as COVID-19 becomes endemic in many areas, positive PCR, serology or exposure may merely represent a concurrent phenomenon, and not necessarily explain the clinical presentation.

Focusing on the GI and myocardial involvement was recently suggested to be useful in differentiating MIS-C from other “look-alike” clinical entities^14^. Our report adds lymphopenia as a useful simple aid to differentiate between MIS-C and KD. This differentiation is not semantic but has therapeutic and prognostic implications. All of our MIS-C patients responded to corticosteroid therapy with 7/10 responding promptly to standard high dose and 3/10 only after IV pulse methylprednisolone was added. The present guidelines for immunomodulatory treatment in MIS-C are mainly derived from real-life experience and from high-quality data on the treatment of KD^10^. These guidelines recommend IVIG and/or corticosteroids, either alone or in combination, as first-line agents in MIS-C. In KD patients, corticosteroids have a role only as adjunctive therapy to IVIG in certain circumstances indicating a high risk for coronary involvement and/or non-response, or as a rescue therapy (usually after failing to respond to a 2nd dose of IVIG)^15^. Therefore, patients who meet criteria for both KD (usually incomplete) and MIS-C, should be treated with IVIG as first line treatment. A recent retrospective, propensity-score matched study found that MIS-C patients that received combined treatment of IVIG plus methylprednisolone had a better course of fever compared with those treated with IVIG alone^16^.

Further support to the concept of KD and MIS-C as separate entities, is provided by the relatively stable rate of KD cases during 2020 compared with 2019 at our two medical centers. If SARS-CoV-2 was a trigger to KD, we probably would have found an increase in the incidence of KD by now.

Our report represents the experience of two centers, located in regions with high COVID-19 incidence rates. As the COVID-19 epidemic continues to spread, there is an urgent need to improve our understanding of this new disease, particularly developing a more accurate diagnostic criteria that also clearly differentiate this entity from KD. Other multicenter control or “real-life” consensus treatment plan (CTP) studies should focus on determining the optimal treatment modality.

## Supplementary Information

Supplementary Legend.

Supplementary Figure S1.
