# Supplementary material for: Characterizing the differences between multisystem inflammatory syndrome in children and Kawasaki disease
Source: Sci Rep. 2021 Jul 5;11:13840. doi: 10.1038/s41598-021-93389-0 (PMC8257717; doi:10.1038/s41598-021-93389-0)
Supplement: Supplementary file 1 — Supplementary Legend. [file 41598_2021_93389_MOESM1_ESM.docx]

Figure S1. Clinical and laboratory characteristics of patients with Kawasaki Disease (KD), multisystem inflammatory syndrome in children (MIS-C) and the overlapping group of patients meeting criteria for both. Percentages of patients are presented for each variable: Age>5 years,

Gastrointestinal (GI) symptoms, Hypotension, lymphocyte count <1500 µL, platelets <150K/µL, left ventricular (LV) dysfunction, valvulitis, pericardial effusion and retrograde aortic diastolic flow.
