# Supplementary figures and images for: Characterizing the differences between multisystem inflammatory syndrome in children and Kawasaki disease
Source: Sci Rep. 2021 Jul 5;11:13840. doi: 10.1038/s41598-021-93389-0 (PMC8257717; doi:10.1038/s41598-021-93389-0)

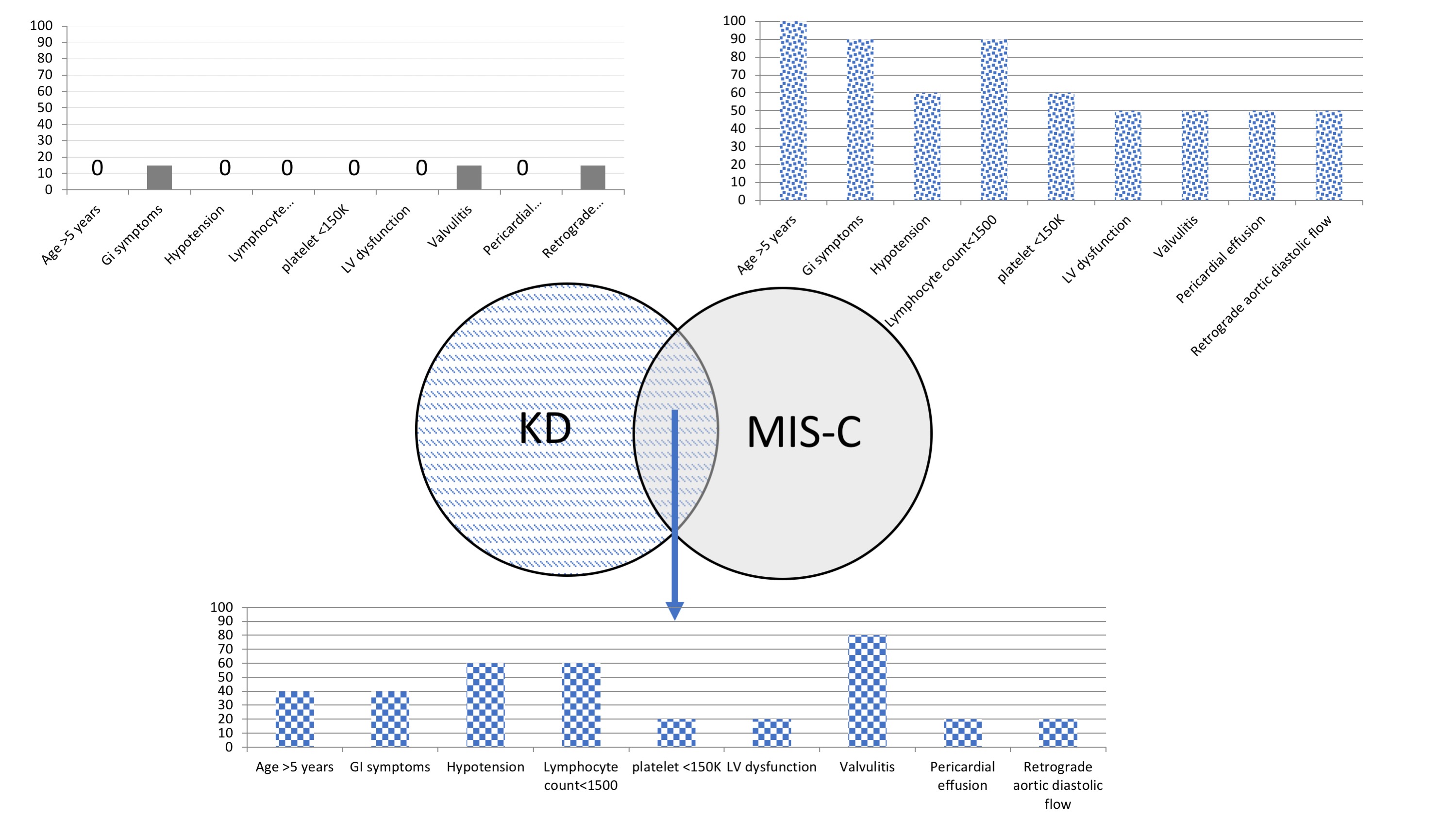

Supplement: Supplementary file 2 — Supplementary Figure S1. [file 41598_2021_93389_MOESM2_ESM.jpg]
